# Brown Rot-Type Fungal Decomposition of Sorghum Bagasse: Variable Success and Mechanistic Implications

**DOI:** 10.1155/2018/4961726

**Published:** 2018-04-03

**Authors:** Gerald N. Presley, Bongani K. Ndimba, Jonathan S. Schilling

**Affiliations:** ^1^Department of Bioproducts and Biosystems Engineering, University of Minnesota, 2004 Folwell Ave. St. Paul, MN 55108, USA; ^2^Agricultural Research Council of South Africa (ARC-Infruitec/Nietvoorbij), Private Bag X5026, Stellenbosch 7599, South Africa; ^3^Department of Biotechnology, University of the Western Cape, Private Bag X17, Bellville 7535, South Africa; ^4^Department of Plant and Microbial Biology, University of Minnesota, 1500 Gortner Ave. St. Paul, MN 55108, USA

## Abstract

Sweet sorghum is a promising crop for a warming, drying African climate, and basic information is lacking on conversion pathways for its lignocellulosic residues (bagasse). Brown rot wood-decomposer fungi use carbohydrate-selective pathways that, when assessed on sorghum, a grass substrate, can yield information relevant to both plant biomass conversion and fungal biology. In testing sorghum decomposition by brown rot fungi (*Gloeophyllum trabeum*,* Serpula lacrymans*), we found that* G. trabeum* readily degraded sorghum, removing xylan prior to removing glucan.* Serpula lacrymans*, conversely, caused little decomposition. Ergosterol (fungal biomarker) and protein levels were similar for both fungi, but* S. lacrymans* produced nearly 4x lower polysaccharide-degrading enzyme specific activity on sorghum than* G. trabeum*, perhaps a symptom of starvation. Linking this information to genome comparisons including other brown rot fungi known to have a similar issue regarding decomposing grasses* (Postia placenta, Fomitopsis pinicola)* suggested that a lack of CE 1 feruloyl esterases as well as low xylanase activity in* S. lacrymans* (3x lower than in* G. trabeum*) may hinder* S. lacrymans*,* P. placenta, *and* F. pinicola *when degrading grass substrates. These results indicate variability in brown rot mechanisms, which may stem from a differing ability to degrade certain lignin-carbohydrate complexes.

## 1. Introduction

Renewable biofuel and bio-based products are an avenue towards energy independence and rural economic growth in southern Africa [1]. In the case of bioethanol in southern Africa, the predominant plant feedstock is water-intensive sugar cane [2], but changes in Sub-Saharan climates are predicted to result in dryer conditions and demand more diverse crop options in order for agriculture to adapt [3]. Sweet stem sorghum* (Sorghum bicolor)* is a native African plant grown for sugar that is more tolerant of arid climates and saline soils [4, 5], making it an attractive alternative bioenergy crop for southern Africa, and its residues after sugar extraction (bagasse) are an attractive biomass crop for many different bio-based product options. The lignocellulose in extracted sorghum bagasse could be utilized for silage or as a feedstock for biofuels and bioproducts, given efficient deconstruction [6]. The sugars locked in sorghum bagasse, however, are more difficult to liberate than those extractable in hot water, requiring harsher pretreatments to liberate fermentable sugars [7].

To better understand how sorghum bagasse could be deconstructed using milder, bio-based techniques, it is logical to study how sorghum is deconstructed by lignocellulose-degrading fungi. For this study, we chose to assess the mechanisms of lignocellulose-degrading “brown rot” fungi, a current research focus due to their carbohydrate-selective extraction pathways and their consolidation of oxidative pretreatments with enzymatic saccharification, two steps that remain separate in industrial processing [8]. In addition to a relevant bioprocessing system, investigating brown rot of sorghum is logical due to the varying abilities to degrade lignin and monocot grasses [9], something that is rarely tested among decomposer fungi associated with wood substrates. Brown rot fungi are polyphyletic [10], and while some characteristics such as early hemicellulose removal are common among brown rot fungi [11, 12], their genomes harbor significant variability [13, 14] and brown rot fungi can be found in a wide variety of niches in nature [15]. This implies variability in decay mechanisms among brown rot fungal clades, a useful model system for probing decay pathways for an understudied feedstock such as sweet stem sorghum bagasse as well as offering a useful context for probing fungal biochemical variability.

For our study, we chose to focus specifically on* Gloeophyllum trabeum* and* Serpula lacrymans* from two distinct clades (Gloeophyllales and Boletales, resp.).* S. lacrymans* has several key genomic distinctions from* G. trabeum*. These include the retention of exoacting cellobiohydrolase (CBH) cellulase genes, but, similar to* Postia placenta*, the absence of carbohydrate esterase family 1 (CE 1), a family containing feruloyl esterases (EC 3.1.1.73) that might be involved in decoupling xylan from lignin, particularly in the decay of grasses [16]. We have also seen on wood substrates (spruce, [17]) that* S. lacrymans* preferentially produced mannanase activity and* G. trabeum* produced higher xylanase activity, despite causing a similar type of decay. Sorghum, like other Poaceae substrates [18], is a xylan-rich substrate that would require* S. lacrymans* to alter its mannanase-dominant glycoside hydrolase profile in order to survive, as well as decoupling xylan from lignin by the hydrolysis of ferulic acid esters. Xylan is a barrier to the majority of the utilizable carbon in plant biomass [19], and failure by fungi to remove it could result in starvation and associated cell autolysis [20].

In this study,* G. trabeum* and* S. lacrymans* were compared for their ability to grow on water-extracted sweet stem sorghum biomass, the bagasse substrate (after extraction) most relevant to sorghum bioconversion. The fungi were grown for 7, 14, and 21 days and the loss of major structural carbohydrates, relative to undegraded sorghum, was measured along with polysaccharide-degrading enzyme activity and ergosterol contents. Decay strategies were compared to previous patterns generated on wood, as well as to other works showing variable decay patterns on other grass substrates.

## 2. Methods

### 2.1. Sorghum Collection and Preparation

Sweet stem sorghum* (Sorghum bicolor)* stalks were grown outside of East London, South Africa, maintained on Agricultural Research Council of South Africa research plots, and were harvested in May 2016. Four stalks were cut into ~2 cm long sections near the top of the stalks (<2 cm diameter). The stalk sections were dried at 70°C and then extracted in water at 90°C with 5 exchanges of fresh water. This approach removes some hemicellulose, along with other more soluble sugars and is a more biotechnologically relevant substrate, as compared to postextracted bagasse. After extraction, the stems were dried at 70°C and stored until further use.

### 2.2. Fungal Cultivation and Decay Microcosms

*Serpula lacrymans* S7.3 and* Gloeophyllum trabeum* ATCC 11539 cultures were maintained on potato dextrose agar. Agar plugs (1 cm dia) from plates colonized by either fungus were used to inoculate modified soil block jars, as previously described [17]. Extracted sorghum sections were vacuum-impregnated with water, sterilized (121°C, 16 psi, 1 hour), and cooled. Four sections were added to each soil block microcosm after allowing the fungus to colonize for 2 weeks, and then the microcosms were incubated at room temperature in the dark for 7, 14, and 21 days. Sorghum sections prepared in parallel but not inoculated were used as control material for characterization.

### 2.3. Protein Extraction, Purification, and Activity Assays

At each timepoint, degraded and nondegraded sorghum discs were chopped into smaller pieces by hand, suspended in 80 ml of extraction buffer (50 mM acetate, 0.5 M NaCl, 0.05% Tween 80, pH 5.0), and then extracted at 4°C with gentle shaking for 24 hours. Two replicate extracts of four pooled sorghum discs at each timepoint were used to generate protein extracts for each fungus. Coarse material was filtered using a polyester mesh, dried at 70°C, and reserved for chemical analysis. The filtrate was centrifuged (4000*g*, 30 min) to remove particulates, filtered through 0.2 *μ*m polyethersulfone (PES) filters, and exchanged into 50 mM citrate buffer pH 5.0 through 10 kDa PES membranes. Protein concentration was determined using a Bio-Rad protein assay kit (Hercules, CA, USA).

Cellulase and hemicellulase specific activities were measured by the dinitrosalicylic acid (DNS) method using solutions of 1.5% carboxymethyl cellulose (endoglucanase), 2% birchwood xylan (xylanase), and 0.5% locust bean gum (mannanase) [21]. Protein extracts were incubated with substrate at 50°C in 50 mM citrate pH 5.0 in triplicate. The absorbance at 540 nm was measured after color development, and reducing sugars were determined as glucose, xylose, and mannose reducing equivalents for endoglucanase, xylanase, and mannanase activities, respectively. Activities for *β*-glucosidase and *β*-xylosidase were determined by measuring the release of 4-nitrophenol (4NP) from 4-nitrophenol-*β*-glucoside (4NPG) and 4-nitrophenol-*β*-xylopyranoside (4NPX), respectively. Reactions were carried out in 10 mM 4NP-substrate in 50 mM citrate, pH 5.0 at 50°C, and were quenched with 2 volumes of 0.2 M Na_2_CO_3_. Absorbance at 400 nm was measured to determine free 4NP.

### 2.4. Ergosterol Extraction and Assay

Total ergosterol was measured to be used as a biomarker for fungal biomass and was extracted from three separate sorghum sections for each fungus at 7, 14, and 21 days of decay and from nondegraded sorghum using established methods [22]. Ergosterol was measured by HPLC using a Phenomenex™ (Torrance, CA, USA) 4 *μ* Hydro-RP 80a column by detection at 282 nm using previously described methods [23].

### 2.5. Sorghum Mass Loss, Density, and Compositional Analyses

Mass loss was determined for sorghum degraded for 7, 14, and 21 days and density of degraded and nondegraded sorghum sections were determined in triplicate by measuring the mass of water displaced by submerging fully hydrated sorghum sections in excess water (g cm^−3^). Degraded and nondegraded sorghum biomass previously extracted for protein were milled to 40 mesh in a Wiley mill. Sorghum powder was hydrolyzed in dilute acid and the concentration of glucan, xylan, and arabinan were measured by HPLC using an Aminex HPX87-P column (Bio-Rad, Hercules, CA) according to standard procedures [24]. The percent loss of each component was calculated from original component mass calculated using average mass losses at each timepoint.

## 3. Results

### 3.1. Decay Rates and Fungal Biomass on Sorghum

*Gloeophyllum trabeum* degraded sorghum more completely than* Serpula lacrymans*, causing nearly 5x greater mass loss (37.8% versus 7.9%, respectively) after 21 days of decay (Table 1). As typical of brown rot, early-stage strength loss was evident and residues were easily crumbled in the hand, more so for sorghum incubated with* G. trabeum*. Despite differences in degradative ability, ergosterol levels, a proxy for fungal biomass, did not differ at equivalent timepoints between the fungi and indicated ample colonization but minimal substrate degradation for* S. lacrymans *(Table 1). The mass of total protein extracted from sorghum biomass was also similar between the two fungi at most timepoints, ranging from 71 to 107 *μ*g cm^−3^ of biomass (Table 1).

### 3.2. Sorghum Carbohydrate Losses

**Figure 1:**
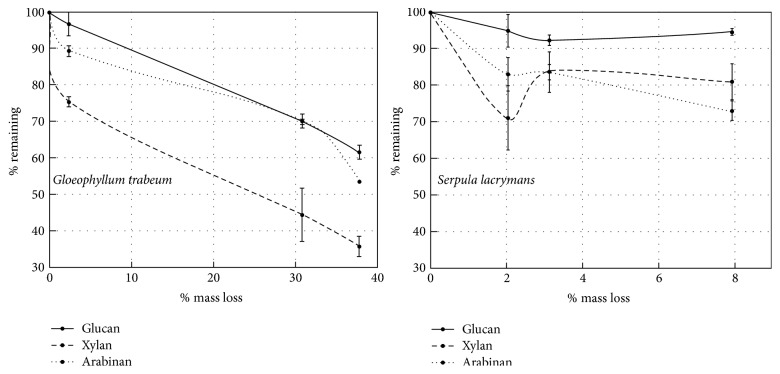
Major structural carbohydrate removal from sorghum biomass over a 21-day progression of decay for* Gloeophyllum trabeum* and* Serpula lacrymans*. Error bars are +/− standard error of three replicate assays of each of the two replicate extracts.

Xylan was removed more rapidly from sorghum than glucan by both species, with 24.5% and 29% xylan loss after 7 days of decay compared to 3.2% and 5% glucan loss over the same period by* G. trabeum* and* S. lacrymans*, respectively (Figure 1). Arabinan loss at 7 days for both species tended to be higher than glucan, but not significantly at 95% confidence (Tukey's HSD, *p* > 0.05). Glucan removal proceeded rapidly in the later stages of decay by* G. trabeum*, but this was not the case for* S. lacrymans*, reaching 38.4% loss by 21 days of decay in the former, and only 5.3% loss in the latter.

In relative terms (% of calculated original polymer mass), xylan was the most labile polysaccharide for* G. trabeum*, and 62.2% of the original xylan was lost after 21 days of decay (Figure 1). In absolute terms (mg of polymer), the mass of glucan and xylan removed by* G. trabeum* did not differ significantly until after 21 days of decay, where the total mass of glucan and xylan lost was 81 mg and 54.7 mg, respectively.* S. lacrymans* removed relatively more xylan and arabinan than glucan by 21 days of decay and did not remove either of the major hemicelluloses preferentially. The mass of polysaccharide components removed over time did not differ significantly from one another for sorghum degraded by* S. lacrymans* indicating no sugar preference.* S. lacrymans* also degraded lower percentages of all components than* G. trabeum*, collectively explaining low mass loss values.

### 3.3. Enzyme Activities

**Figure 2:**
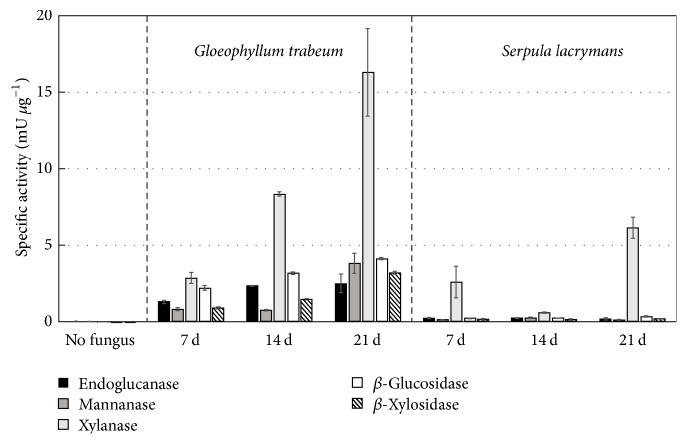
Endoglucanase, Xylanase, Mannanase, *β*-glucosidase, and *β*-xylosidase specific activities of protein extracts from sorghum sections degraded by* Gloeophyllum trabeum* and* Serpula lacrymans* for 7, 14, and 21 days and undegraded sorghum biomass. Error bars are +/− standard error of three replicate assays of each of two replicate extracts.

In* G. trabeum* extracts, most enzyme specific activities increased from early to late stages of decay except for endoglucanase activity, which remained flat throughout decay stages (Figure 2) (*p* > 0.05, Tukey's HSD). In* S. lacrymans*, enzyme activities tended to be much lower, despite similar levels of total protein to* G. trabeum*. All measured activities in* S. lacrymans*, except for xylanase at 21 days, were not significantly above nondegraded controls (*p* > 0.05, Tukey's HSD). In line with patchy growth patterns observed in* S. lacrymans*, xylanase activity at 7 days was highly variable between the two replicate extractions, with activity from one extract matching that of nondegraded controls. For both fungi, xylanase was the highest polysaccharide-degrading enzyme activity. Despite this, *β*-xylosidase (BXL) activity was lower than *β*-glucosidase (BGL) activity in* G. trabeum* extracts.

## 4. Discussion

In this study, the brown rot fungus* Gloeophyllum trabeum* (Gloeophyllales) effectively degraded sorghum while the fungus* Serpula lacrymans* (Boletales) struggled to release carbohydrates from the substrate. This variability in apparent recalcitrance of the sorghum substrate is an important result, but the variability between the fungi is also informative. Brown rot fungi are often assumed to have a preference for conifer substrates (gymnosperms) rather than angiosperm substrates in nature, and it is notable that* G. trabeum* is commonly found on the wood of both conifers and angiosperms [15]. This lack of substrate specificity for* G. trabeum* might partially explain its superiority to* S. lacrymans* on sorghum, although these substrate associations are poorly understood and often do not persist outside of a natural setting. Wood-degrading fungi that are host-specific in the field are often capable of growing and deconstructing a wider range of substrates when grown in culture [25, 26]. These results also support a broader variability in fungal deconstruction pathways among the brown rot types.

Successful growth on xylan-rich sorghum by* G. trabeum* may be due in part to high xylanase activity, a trait that would also enable decay of angiosperm wood whose hemicellulose is dominated by xylan. Both of our test fungi initiated the decomposition process by removing xylan, reflecting the protective role of xylan in shielding glucan from enzymatic hydrolysis [19]. Similar to the distinction between these fungi previously on spruce wood [17], xylanase specific activities were approximately 3x higher on average for* G. trabeum* than for* S. lacrymans*. It is plausible that* G. trabeum* has more responsive xylanase induction than* S. lacrymans*, stimulated by endoxylanase-produced xylo-, di-, and oligosaccharides as in other fungal species [27, 28]. In* G. trabeum*, BXL activity after 7 days was considerably lower than xylanase activity, implying that the majority of xylanase activity at the early decay stages is due to endo-acting xylanases required to liberate *β*-xylosidase substrates [29].

Differences in the degradative capacity of brown rot fungi may also be explained by the number and types of lignocellulose-degrading genes in their genomes that would be effective on a grass substrate. Along with* S. lacrymans*,* Postia placenta* and* Fomitopsis pinicola* have been shown to be ineffective in degrading a wide range of Poaceae substrates [9]. Grasses such as corn stover contain hemicelluloses that are similar to sorghum, primarily consisting of glucuronoarabinoxylan with ferulic acid ester linkages between xylan and lignin [30]. Among the types of genes involved in the degradation of grass polysaccharides, CE 1 feruloyl esterases (FAE) (EC 3.1.1.73) are absent in the genomes of* P. placenta*,* F. pinicola*, and* S. lacrymans* (Table 2) [14]. Absence of FAE activity has also been demonstrated in cultures of* Postia placenta*, indicating a minimal capacity to hydrolyze ferulic acid esters in lignocellulose [31]. In contrast,* G. trabeum* is known to effectively degrade Poaceae substrates [9] and possesses a CE 1 that is actively secreted on wood [17], suggesting CE 1 proteins could be the basis of the difference in degradative abilities observed in this study.

Despite differences in degradative ability, both fungi produced similar amounts of ergosterol, a proxy for total fugal biomass [22], as well as similar amounts of secreted protein on sorghum when compared at equivalent timepoints. Ergosterol/protein ratios (E/P) for the two fungi did not differ significantly at any decay stage, unlike previous observations on spruce where* G. trabeum* produced relatively more protein and less ergosterol (lower E/P) than* S. lacrymans* [17]. These patterns in E/P are likely explained by increased protein investments on sorghum, particularly for* S. lacrymans*; however, polysaccharide-degrading enzyme specific activity was on average 13x lower on sorghum biomass compared to spruce, whereas the same discrepancy was less than 4x lower for* G. trabeum*. The differences in specific activity may be linked to the relative inability of* S. lacrymans* to metabolize sorghum, causing C-starvation, cell autolysis, and the dilution of secreted protein with cellular protein as seen in C-starved cultures of* Paxillus involutus *and* Aspergillus niger* [20, 32]. This indicates that quantifying protein investments as a “trait,” including nitrogen-use efficiencies, might yield very different trait values for the same wood-degrading fungi, depending on the substrates used to calculate these values.

## 5. Conclusion

This study highlights differences in the biodegradative ability and decay mechanisms among phylogenetically disparate brown rot fungi.* S. lacrymans*, like some other brown rot fungi, may have general difficulty degrading grass substrates. The more generalist substrate associations of* G. trabeum*, however, may impart an ability to degrade grasses. The discrepancies in sorghum-degrading ability may be linked to the presence of fungal genes coding for enzymes that target xylan-lignin bonds and xylan, itself. This would be useful to explore in other brown rot clades, as well, and may help explain substrate-specificities among wood-degrading fungi.

## Figures and Tables

**Table 1:** Ergosterol (E), protein (P), and ergosterol/protein ratios (E/P) for sorghum sections degraded by *Gloeophyllum trabeum* and *Serpula lacrymans *for 7, 14, and 21 days. Protein and ergosterol levels are standardized to cm^3^ of biomass.

	*Gloeophyllum trabeum*				*Serpula lacrymans *			
	Mass loss%^a^	E *µ*g cm^−3^^a^	P *µ*g cm^−3^^b^	E/P	Mass loss%	E *µ*g cm^−3^	P *µ*g cm^−3^	E/P
7 days	2.4 (1.8)	73.3 (44.5)	98.1 (30.8)	0.7	2.0 (1.0)	76.3 (60.8)	107.6 (26.4)	0.7
14 days	30.8 (5.1)	205.0 (59.1)	98.5 (31.1)	2.1	3.1 (1.2)	73.0 (54.2)	71.3 (12.7)	1.0
21 days	37.8 (3.7)	215.0 (33.9)	78.3 (16.5)	2.7	7.9 (3.6)	235.6 (71.0)	74.6 (7.1)	3.2
Sorghum Avg	23.7 (18.7)	164.4 (79.1)	94.5 (22.6)	1.9	4.4 (3.1)	128.3 (93.0)	112.5 (21.6)	1.6
Spruce Avg^c^	N/A	99.8 (31.3)	14.6 (3.5)	6.7	N/A	188.8 (53.6)	5.0 (1.3)	38.6

^a^Standard deviation of three biological replicates is shown in parentheses; ^b^standard deviation of three assays of an extract of 8 sorghum discs is shown in parentheses; ^c^values adapted from Presley and Schilling 2017.

**Table 2:** Number of copies of gene families with activity on glucuronoarabinoxylan, cellulose, and mixed linkage glucan present in the genomes of seven brown rot fungi. The major taxonomic affiliations are shown, SL, *Serpula lacrymans*, CP, *Coniophora puteana*, WC, *Wolfiporia cocos*, PP, *Postia placenta*, FP, *Fomitopsis pinicola*, GT, *Gloeophyllum trabeum*, DP, *Dacryopinax sp.*

Relevant Activity^1^	Family^2^	Brown rot species^3^						
		Boletales		Polyporales			Gloeophyllales	Dacrymycetes
		CP	SL	PP	FP	WC	GT	DP
AXE/FE	CE 1	0	0	0	0	0	1	0
AXE	CE 4	9	6	2	3	4	5	11
AXE	CE 5	1	0	0	0	0	0	0
4MGU	CE 15	0	0	1	1	1	1	1
AE	CE 16	7	4	4	8	6	7	3
BGL/BXL/BGU	GH 1	3	3	2	2	1	5	1
BGU/ABF	GH 2	5	3	3	4	3	4	3
BGL/BXL/ABF/B3G/B4G	GH 3	13	10	6	12	8	11	9
EGL/EXL/BGL/B3G/EBG	GH 5	21	21	17	19	18	19	24
EGL/CBHII	GH 6	2	1	0	0	0	0	0
EGL/CBHI/E34G	GH 7	2	0	0	0	0	0	0
EGL/BGL/E34G/EBG/CBH	GH 9	1	1	0	0	0	1	1
EXL	GH 10	3	1	3	2	4	3	3
EGL/E34G	GH 12	4	2	2	2	2	2	1
E3G/E34G	GH 16	24	20	24	28	19	29	14
E3G/E34G	GH 17	4	2	2	3	2	2	3
E34G	GH 26	0	0	0	0	0	0	4
EXG/BGL/BGU/BXL	GH 30	7	2	3	10	2	3	4
BXL/ABF/EXL	GH 43	6	2	1	7	1	6	5
EGL	GH 45	1	0	0	1	0	1	1
EGL/EXL/BXL/AAF	GH 51	3	1	1	4	4	4	2
E3G/B3G	GH 55	5	6	3	3	3	2	2
EGL/EXG	GH 74	0	1	0	0	0	1	0
BGU	GH 79	4	6	2	3	3	6	7
E3G	GH 81	1	0	0	0	0	1	0
ABF	GH 93	1	0	0	0	0	0	0
AGU	GH 115	2	1	1	1	2	2	2
E3G	GH 128	9	5	5	4	2	6	2
E346G	GH 131	2	2	0	1	0	1	1

^1^Each abbreviation represents an EC number indicating the specificity of an enzyme in each family. 4MGU, 4-O-methyl-glucuronyl methylesterase (EC 3.1.1.-), ABF, *α*-arabinofuranosidase (EC 3.2.1.55), AE, acetylesterase (EC 3.1.1.6), AXH, arabinoxylan arabinofuranohydrolase (EC 3.2.1.55), AXE, acetyl xylan esterase (EC 3.1.1.72), AGU, *α*-glucuronidase (EC 3.2.1.131), B3G, *β*-1,3-glucosidase (EC 3.2.1.58), B4G, *β*-1,4-glucosidase (EC 3.2.1.74), BGL, *β*-glucosidase (EC 3.2.1.21), BGU, *β*-glucuronidase (EC 3.2.1.31), BXL, *β*-xylosidase (EC 3.2.1.37), CBHII, cellobiohydrolase (EC 3.2.1.91), CBHI, cellobiohydrolase reducing end (EC 3.2.1.176), E34G, endo-*β*-1,3-1,4-glucanase (EC 3.2.1.73/3.2.1.6), E364G, exo-*β*-1,3-1,6 and endo-*β*-1,4-glucanase (EC 3.2.1.-), E3G, endo-*β*-1,3-glucanase (EC 3.2.1.39), EXG, exo-*β*-1,4-glucanase (EC 3.2.1.74), EGL, endo-*β*-1,4-glucanase (EC 3.2.1.4), EXL, endo-*β*-1,4-xylanase (EC 3.2.1.8), FE, feruloyl esterase (3.1.1.73); ^2^CE, carbohydrate esterase, GH, glycoside hydrolase; ^3^gene counts are adapted from [14].
